# (5-Amino­isophthalato-κ*N*)triaqua­(1,10-phenanthroline-κ^2^ *N*,*N*′)nickel(II) trihydrate

**DOI:** 10.1107/S1600536811008919

**Published:** 2011-03-15

**Authors:** Han Huang, Kou-Lin Zhang, Seik Weng Ng

**Affiliations:** a College of Chemistry and Chemical Engineering, Yangzhou University, Yangzhou 225002, People’s Republic of China; b Department of Chemistry, University of Malaya, 50603 Kuala Lumpur, Malaysia

## Abstract

The Ni^II^ atom in the title compound, [Ni(C_8_H_5_NO_4_)(C_12_H_8_N_2_)(H_2_O)_3_]·3H_2_O, is six-coordinated in an NiN_3_O_3_ octa­hedral geometry. The triply water-coordinated Ni^II^ atom is chelated by the phenantroline ligand and is additionally coordinated by the amino group of the 5-amino­isophtalate anion. The anion, the coordinated and the uncoordinated water mol­ecules inter­act through an extensive O—H⋯O and N—H⋯O hydrogen-bonding network, generating a three-dimensional cage-like network.

## Related literature

For the isotypic Co^II^ analog, see: Zhang *et al.* (2010 ▶).
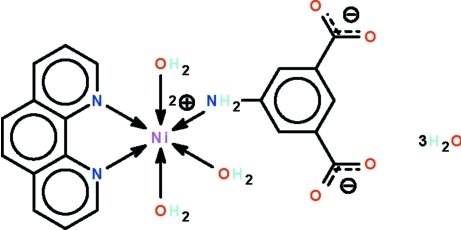


## Experimental

### 

#### Crystal data

[Ni(C_8_H_5_NO_4_)(C_12_H_8_N_2_)(H_2_O)_3_]·3H_2_O
*M* *_r_* = 526.14
Monoclinic, 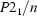

*a* = 10.1039 (10) Å
*b* = 13.9448 (14) Å
*c* = 16.4237 (16) Å
β = 95.522 (1)°
*V* = 2303.3 (4) Å^3^
*Z* = 4
Mo *K*α radiation
μ = 0.90 mm^−1^
*T* = 295 K
0.13 × 0.12 × 0.10 mm

#### Data collection

Bruker APEXII area-detector diffractometer
Absorption correction: multi-scan (*SADABS*; Sheldrick, 1996 ▶) *T*_min_ = 0.892, *T*_max_ = 0.915
20080 measured reflections
5258 independent reflections
3702 reflections with *I* > 2σ(*I*)
*R*_int_ = 0.092

#### Refinement

*R*[*F* ^2^ > 2σ(*F* ^2^)] = 0.037
*wR*(*F* ^2^) = 0.081
*S* = 0.89
5258 reflections
363 parameters
14 restraints
H atoms treated by a mixture of independent and constrained refinement
Δρ_max_ = 0.35 e Å^−3^
Δρ_min_ = −0.55 e Å^−3^

### 

Data collection: *APEX2* (Bruker, 2004 ▶); cell refinement: *SAINT* (Bruker, 2004 ▶); data reduction: *SAINT*; method used to solve structure: atomic coordinates taken from an isotypic structure (Zhang *et al.*, 2010 ▶); program(s) used to refine structure: *SHELXL97* (Sheldrick, 2008 ▶); molecular graphics: *X-SEED* (Barbour, 2001 ▶); software used to prepare material for publication: *publCIF* (Westrip, 2010 ▶).

## Supplementary Material

Crystal structure: contains datablocks global, I. DOI: 10.1107/S1600536811008919/wm2464sup1.cif

Structure factors: contains datablocks I. DOI: 10.1107/S1600536811008919/wm2464Isup2.hkl

Additional supplementary materials:  crystallographic information; 3D view; checkCIF report

## Figures and Tables

**Table 1:** Selected bond lengths (Å)

Ni1—O1*w*	2.0408 (16)
Ni1—N2	2.0800 (16)
Ni1—O2*w*	2.0797 (14)
Ni1—N3	2.0883 (16)
Ni1—O3*w*	2.0965 (15)
Ni1—N1	2.1409 (18)

**Table 2:** Hydrogen-bond geometry (Å, °)

*D*—H⋯*A*	*D*—H	H⋯*A*	*D*⋯*A*	*D*—H⋯*A*
O1*w*—H1*w*1⋯O6*w*^i^	0.84 (1)	1.91 (1)	2.747 (2)	177 (3)
O1*w*—H1*w*2⋯O2^ii^	0.84 (1)	1.82 (1)	2.654 (2)	180 (2)
O2*w*—H2*w*1⋯O5*w*^i^	0.85 (1)	1.94 (1)	2.781 (2)	173 (3)
O2*w*—H2*w*2⋯O4^iii^	0.85 (1)	1.98 (1)	2.832 (2)	178 (3)
O3*w*—H3*w*1⋯O5*w*^iv^	0.84 (1)	2.16 (2)	2.914 (3)	148 (3)
O3*w*—H3*w*2⋯O3^ii^	0.85 (1)	1.88 (1)	2.721 (2)	173 (3)
O4*w*—H4*w*1⋯O6*w*^v^	0.85 (1)	1.97 (1)	2.817 (3)	171 (3)
O4*w*—H4*w*2⋯O2^iv^	0.85 (1)	2.16 (2)	2.915 (3)	149 (3)
O5*w*—H5*w*1⋯O1	0.85 (1)	1.90 (1)	2.726 (3)	163 (3)
O5*w*—H5*w*2⋯O3^vi^	0.84 (1)	1.91 (1)	2.716 (2)	159 (3)
O6*w*—H6*w*1⋯O1	0.85 (1)	1.83 (1)	2.678 (2)	177 (3)
O6*w*—H6*w*2⋯O4^iii^	0.85 (1)	1.95 (1)	2.791 (2)	176 (3)
N1—H1⋯O4*w*	0.85 (1)	2.08 (1)	2.928 (3)	173 (2)
N1—H2⋯O4^iii^	0.85 (1)	2.30 (1)	3.116 (2)	162 (2)

Symmetry codes: (i) 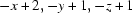; (ii) 
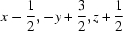
; (iii) 
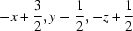
; (iv) 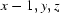; (v) 
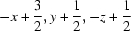
; (vi) 
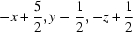
.

**Fig. 1.**
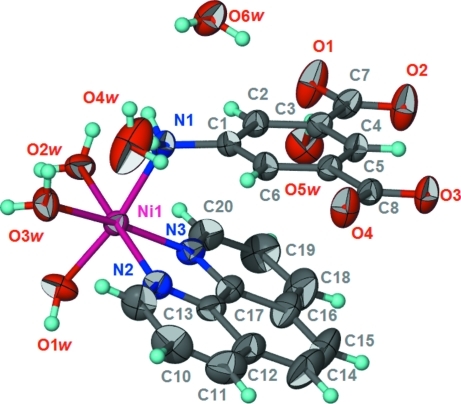
The molecular entities of (I) with atom labelling and displacement parameters at the 70% probability level; hydrogen atoms are shown as spheres of arbitrary radius.

## supplementary crystallographic information

### Comment

We reported the structure of [Co(C_8_H_5_NO_4_)(C_12_H_8_N_2_)(H_2_O)_3_]^.^3H_2_O], whose 5-aminoisophthlate dianion binds only through the neutral amino donor site. The coordinated water molecules comprise the *fac* points of the NiN_3_O_3_ octahedron (Zhang *et al.*, 2010). The nickel analog (Scheme I) is isotypic. The dianion, the coordinated and the lattice water molecules interact through hydrogen bonds (Table 1) to furnish a tightly-held, three-dimensional network. Pairs of phenanthroline units show π···π interactions about a center-of-inversion at a distance of *ca* 3.5 Å.

### Experimental

Nickel nitrate hexahydrate (0.048 g, 0.165 mmol) dissolved in water (5 ml) was added to a mixture of 5-aminoisophthalic acid (0.030 g, 0.165 mmol) and sodium hydroxide (0.013 g, 0.330 mmol) dissolved in water (5 ml). To this solution was added 1,10-phenanthroline (0.033 g, 0.165 mmol) dissolved in methanol (10 ml). The mixture was filtered and set aside for the growth of green crystals.

### Refinement

Hydrogen atoms were placed in calculated positions (C—H 0.93 Å) and were included in the refinement in the riding model approximation, with *U*_iso_(H) set to 1.2*U*_eq_(C). The amino and water bound H-atoms were located in difference Fourier maps, and were refined with a distance restraint of N–H = O–H = 0.85 (1) Å. Their temperature factors were freely refined.

### Crystal data

**Table:**

[Ni(C_8_H_5_NO_4_)(C_12_H_8_N_2_)(H_2_O)_3_]·3H_2_O	*F*(000) = 1096
*M**_r_* = 526.14	*D*_x_ = 1.517 Mg m^−^^3^
Monoclinic, *P*2_1_/*n*	Mo *K*α radiation, λ = 0.71073 Å
Hall symbol: -P 2yn	Cell parameters from 5121 reflections
*a* = 10.1039 (10) Å	θ = 2.3–26.3°
*b* = 13.9448 (14) Å	µ = 0.90 mm^−^^1^
*c* = 16.4237 (16) Å	*T* = 295 K
β = 95.522 (1)°	Block, green
*V* = 2303.3 (4) Å^3^	0.13 × 0.12 × 0.10 mm
*Z* = 4	

### Data collection

**Table:**

Bruker APEXII area-detector diffractometer	5258 independent reflections
Radiation source: fine-focus sealed tube	3702 reflections with *I* > 2σ(*I*)
graphite	*R*_int_ = 0.092
φ and ω scans	θ_max_ = 27.5°, θ_min_ = 1.9°
Absorption correction: multi-scan (*SADABS*; Sheldrick, 1996)	*h* = −13→12
*T*_min_ = 0.892, *T*_max_ = 0.915	*k* = −18→18
20080 measured reflections	*l* = −21→20

### Refinement

**Table:**

Refinement on *F*^2^	Primary atom site location: structure-invariant direct methods
Least-squares matrix: full	Secondary atom site location: difference Fourier map
*R*[*F*^2^ > 2σ(*F*^2^)] = 0.037	Hydrogen site location: inferred from neighbouring sites
*wR*(*F*^2^) = 0.081	H atoms treated by a mixture of independent and constrained refinement
*S* = 0.89	*w* = 1/[σ^2^(*F*_o_^2^) + (0.0347*P*)^2^] where *P* = (*F*_o_^2^ + 2*F*_c_^2^)/3
5258 reflections	(Δ/σ)_max_ = 0.001
363 parameters	Δρ_max_ = 0.35 e Å^−^^3^
14 restraints	Δρ_min_ = −0.55 e Å^−^^3^

### Fractional atomic coordinates and isotropic or equivalent isotropic displacement parameters (Å^2^)

**Table:**

	*x*	*y*	*z*	*U*_iso_*/*U*_eq_	
Ni1	0.74294 (2)	0.686043 (16)	0.465623 (15)	0.02405 (8)	
O1	1.15737 (15)	0.53335 (11)	0.26472 (11)	0.0506 (5)	
O2	1.22244 (15)	0.64153 (11)	0.18022 (11)	0.0484 (4)	
O3	0.91901 (14)	0.91569 (10)	0.09761 (9)	0.0368 (4)	
O4	0.72199 (14)	0.92528 (10)	0.14342 (10)	0.0413 (4)	
O1w	0.76412 (16)	0.70612 (11)	0.58928 (10)	0.0360 (4)	
H1w1	0.8275 (18)	0.6793 (16)	0.6169 (14)	0.054 (8)*	
H1w2	0.751 (2)	0.7541 (11)	0.6177 (12)	0.044 (7)*	
O2w	0.79265 (16)	0.54505 (10)	0.49643 (10)	0.0331 (3)	
H2w1	0.743 (2)	0.5271 (18)	0.5323 (12)	0.059 (9)*	
H2w2	0.787 (3)	0.5091 (17)	0.4543 (11)	0.074 (10)*	
O3w	0.53887 (15)	0.66633 (12)	0.47353 (11)	0.0370 (4)	
H3w1	0.493 (3)	0.642 (2)	0.4334 (14)	0.097 (12)*	
H3w2	0.507 (3)	0.6419 (18)	0.5148 (11)	0.071 (9)*	
O4w	0.4426 (2)	0.71882 (19)	0.28820 (16)	0.0763 (7)	
H4w1	0.449 (3)	0.7708 (14)	0.2618 (18)	0.090 (12)*	
H4w2	0.403 (3)	0.681 (2)	0.2532 (18)	0.111 (16)*	
O5w	1.36084 (19)	0.52842 (12)	0.38634 (12)	0.0482 (4)	
H5w1	1.309 (2)	0.5233 (18)	0.3428 (10)	0.055 (9)*	
H5w2	1.4241 (18)	0.4915 (16)	0.3787 (16)	0.061 (9)*	
O6w	1.03103 (17)	0.37826 (11)	0.31434 (11)	0.0426 (4)	
H6w1	1.069 (3)	0.4278 (14)	0.2974 (18)	0.088 (11)*	
H6w2	0.9539 (14)	0.3894 (18)	0.3281 (16)	0.062 (9)*	
N1	0.70709 (18)	0.64237 (12)	0.34047 (11)	0.0268 (4)	
H1	0.6280 (12)	0.6600 (15)	0.3259 (14)	0.042 (7)*	
H2	0.708 (2)	0.5815 (7)	0.3415 (14)	0.044 (7)*	
N2	0.71486 (16)	0.83087 (11)	0.43901 (11)	0.0306 (4)	
N3	0.93864 (16)	0.72811 (11)	0.45417 (11)	0.0282 (4)	
C1	0.79789 (19)	0.67558 (13)	0.28500 (12)	0.0247 (4)	
C2	0.91676 (19)	0.62745 (13)	0.27875 (12)	0.0266 (4)	
H2A	0.9348	0.5716	0.3087	0.032*	
C3	1.00920 (19)	0.66171 (13)	0.22835 (12)	0.0257 (4)	
C4	0.98077 (19)	0.74537 (13)	0.18383 (13)	0.0265 (4)	
H4	1.0420	0.7689	0.1501	0.032*	
C5	0.86192 (19)	0.79422 (13)	0.18922 (12)	0.0248 (4)	
C6	0.77066 (19)	0.75923 (13)	0.24048 (12)	0.0263 (4)	
H6	0.6915	0.7919	0.2449	0.032*	
C7	1.1392 (2)	0.60854 (14)	0.22376 (14)	0.0301 (5)	
C8	0.83267 (19)	0.88458 (13)	0.14050 (13)	0.0260 (4)	
C9	0.6034 (2)	0.88175 (16)	0.43450 (14)	0.0418 (6)	
H9	0.5250	0.8512	0.4450	0.050*	
C10	0.5988 (3)	0.97908 (17)	0.41470 (16)	0.0499 (7)	
H10	0.5190	1.0125	0.4130	0.060*	
C11	0.7119 (3)	1.02457 (16)	0.39797 (15)	0.0501 (7)	
H11	0.7097	1.0895	0.3852	0.060*	
C12	0.8322 (2)	0.97357 (15)	0.40000 (15)	0.0415 (6)	
C13	0.8284 (2)	0.87628 (14)	0.42274 (13)	0.0302 (5)	
C14	0.9557 (3)	1.01401 (18)	0.38102 (17)	0.0592 (8)	
H14	0.9587	1.0781	0.3657	0.071*	
C15	1.0675 (3)	0.96117 (19)	0.38490 (18)	0.0606 (8)	
H15	1.1459	0.9889	0.3708	0.073*	
C16	1.0679 (2)	0.86237 (17)	0.41062 (16)	0.0447 (6)	
C17	0.9485 (2)	0.82052 (14)	0.42931 (13)	0.0309 (5)	
C18	1.1817 (2)	0.80332 (19)	0.41870 (18)	0.0561 (7)	
H18	1.2631	0.8269	0.4055	0.067*	
C19	1.1720 (2)	0.71140 (19)	0.44591 (17)	0.0518 (7)	
H19	1.2472	0.6727	0.4530	0.062*	
C20	1.0490 (2)	0.67576 (16)	0.46297 (14)	0.0376 (5)	
H20	1.0438	0.6129	0.4812	0.045*	

### Atomic displacement parameters (Å^2^)

**Table:**

	*U*^11^	*U*^22^	*U*^33^	*U*^12^	*U*^13^	*U*^23^
Ni1	0.02322 (14)	0.02102 (12)	0.02825 (15)	0.00025 (10)	0.00421 (10)	−0.00008 (11)
O1	0.0315 (9)	0.0409 (9)	0.0806 (13)	0.0094 (7)	0.0107 (9)	0.0334 (9)
O2	0.0349 (9)	0.0411 (9)	0.0730 (12)	0.0125 (7)	0.0248 (8)	0.0254 (9)
O3	0.0359 (8)	0.0326 (8)	0.0437 (10)	0.0020 (6)	0.0139 (7)	0.0150 (7)
O4	0.0363 (9)	0.0308 (8)	0.0594 (11)	0.0116 (7)	0.0170 (8)	0.0170 (7)
O1w	0.0397 (9)	0.0367 (9)	0.0307 (9)	0.0105 (7)	−0.0013 (8)	−0.0102 (7)
O2w	0.0453 (10)	0.0239 (7)	0.0302 (9)	−0.0001 (7)	0.0043 (8)	−0.0023 (7)
O3w	0.0277 (8)	0.0503 (10)	0.0339 (10)	−0.0067 (7)	0.0073 (8)	0.0004 (8)
O4w	0.0468 (12)	0.0806 (16)	0.0972 (19)	−0.0116 (12)	−0.0149 (12)	0.0407 (15)
O5w	0.0492 (12)	0.0439 (10)	0.0510 (12)	0.0107 (9)	0.0022 (10)	0.0044 (9)
O6w	0.0340 (9)	0.0325 (8)	0.0620 (12)	0.0006 (7)	0.0077 (9)	0.0046 (8)
N1	0.0275 (10)	0.0246 (9)	0.0289 (10)	−0.0008 (7)	0.0050 (8)	0.0054 (8)
N2	0.0292 (9)	0.0276 (9)	0.0351 (10)	0.0039 (7)	0.0040 (8)	0.0003 (7)
N3	0.0255 (9)	0.0250 (8)	0.0339 (10)	0.0011 (7)	0.0026 (8)	−0.0019 (7)
C1	0.0277 (10)	0.0246 (10)	0.0220 (10)	−0.0023 (8)	0.0035 (8)	0.0005 (8)
C2	0.0298 (11)	0.0225 (9)	0.0272 (12)	0.0005 (8)	0.0020 (9)	0.0038 (8)
C3	0.0247 (11)	0.0233 (9)	0.0291 (12)	0.0007 (8)	0.0023 (9)	0.0010 (8)
C4	0.0255 (10)	0.0250 (9)	0.0297 (11)	−0.0002 (8)	0.0062 (9)	0.0047 (8)
C5	0.0254 (10)	0.0220 (9)	0.0273 (11)	0.0002 (7)	0.0036 (9)	0.0016 (8)
C6	0.0251 (10)	0.0251 (10)	0.0294 (12)	0.0047 (8)	0.0069 (9)	0.0023 (8)
C7	0.0251 (11)	0.0248 (10)	0.0400 (13)	0.0003 (8)	0.0013 (10)	0.0046 (9)
C8	0.0274 (11)	0.0218 (9)	0.0293 (12)	0.0009 (8)	0.0052 (9)	0.0020 (8)
C9	0.0379 (13)	0.0374 (12)	0.0502 (16)	0.0104 (10)	0.0054 (11)	0.0036 (11)
C10	0.0548 (16)	0.0411 (13)	0.0532 (17)	0.0242 (12)	0.0026 (13)	0.0047 (12)
C11	0.0728 (19)	0.0283 (12)	0.0472 (16)	0.0087 (12)	−0.0040 (14)	0.0060 (11)
C12	0.0558 (15)	0.0263 (11)	0.0416 (15)	−0.0030 (10)	−0.0001 (12)	0.0076 (10)
C13	0.0370 (12)	0.0245 (10)	0.0287 (12)	−0.0019 (9)	0.0005 (10)	−0.0008 (9)
C14	0.070 (2)	0.0360 (13)	0.070 (2)	−0.0163 (13)	0.0022 (16)	0.0167 (13)
C15	0.0546 (17)	0.0536 (16)	0.074 (2)	−0.0244 (14)	0.0083 (15)	0.0153 (15)
C16	0.0361 (13)	0.0468 (14)	0.0511 (16)	−0.0122 (11)	0.0034 (12)	0.0046 (12)
C17	0.0296 (11)	0.0293 (10)	0.0336 (12)	−0.0043 (9)	0.0023 (9)	−0.0005 (9)
C18	0.0264 (13)	0.0691 (18)	0.074 (2)	−0.0113 (12)	0.0101 (13)	0.0005 (15)
C19	0.0266 (13)	0.0561 (16)	0.072 (2)	0.0056 (11)	0.0036 (13)	−0.0040 (14)
C20	0.0308 (12)	0.0355 (11)	0.0457 (14)	0.0054 (9)	0.0000 (11)	−0.0018 (10)

### Geometric parameters (Å, °)

**Table:**

Ni1—O1w	2.0408 (16)	C1—C2	1.388 (3)
Ni1—N2	2.0800 (16)	C2—C3	1.391 (3)
Ni1—O2w	2.0797 (14)	C2—H2A	0.9300
Ni1—N3	2.0883 (16)	C3—C4	1.392 (3)
Ni1—O3w	2.0965 (15)	C3—C7	1.516 (3)
Ni1—N1	2.1409 (18)	C4—C5	1.391 (3)
O1—C7	1.250 (2)	C4—H4	0.9300
O2—C7	1.244 (2)	C5—C6	1.395 (3)
O3—C8	1.250 (2)	C5—C8	1.507 (3)
O4—C8	1.259 (2)	C6—H6	0.9300
O1w—H1w1	0.84 (1)	C9—C10	1.395 (3)
O1w—H1w2	0.84 (1)	C9—H9	0.9300
O2w—H2w1	0.85 (1)	C10—C11	1.357 (3)
O2w—H2w2	0.85 (1)	C10—H10	0.9300
O3w—H3w1	0.84 (1)	C11—C12	1.406 (3)
O3w—H3w2	0.85 (1)	C11—H11	0.9300
O4w—H4w1	0.85 (1)	C12—C13	1.409 (3)
O4w—H4w2	0.85 (1)	C12—C14	1.431 (3)
O5w—H5w1	0.85 (1)	C13—C17	1.437 (3)
O5w—H5w2	0.84 (1)	C14—C15	1.346 (4)
O6w—H6w1	0.85 (1)	C14—H14	0.9300
O6w—H6w2	0.85 (1)	C15—C16	1.441 (3)
N1—C1	1.431 (2)	C15—H15	0.9300
N1—H1	0.85 (1)	C16—C17	1.401 (3)
N1—H2	0.85 (1)	C16—C18	1.410 (3)
N2—C9	1.327 (3)	C18—C19	1.364 (4)
N2—C13	1.359 (3)	C18—H18	0.9300
N3—C20	1.329 (2)	C19—C20	1.392 (3)
N3—C17	1.358 (2)	C19—H19	0.9300
C1—C6	1.390 (3)	C20—H20	0.9300
			
O1w—Ni1—N2	94.30 (7)	C5—C4—H4	119.6
O1w—Ni1—O2w	83.56 (6)	C4—C5—C6	119.46 (17)
N2—Ni1—O2w	173.68 (7)	C4—C5—C8	120.15 (17)
O1w—Ni1—N3	92.38 (7)	C6—C5—C8	120.39 (17)
N2—Ni1—N3	79.60 (6)	C1—C6—C5	120.14 (18)
O2w—Ni1—N3	94.52 (6)	C1—C6—H6	119.9
O1w—Ni1—O3w	88.05 (7)	C5—C6—H6	119.9
N2—Ni1—O3w	91.45 (6)	O2—C7—O1	123.19 (19)
O2w—Ni1—O3w	94.41 (6)	O2—C7—C3	118.99 (17)
N3—Ni1—O3w	171.05 (6)	O1—C7—C3	117.82 (19)
O1w—Ni1—N1	170.49 (6)	O3—C8—O4	122.37 (18)
N2—Ni1—N1	93.87 (7)	O3—C8—C5	118.53 (17)
O2w—Ni1—N1	88.81 (6)	O4—C8—C5	119.09 (17)
N3—Ni1—N1	93.88 (7)	N2—C9—C10	122.8 (2)
O3w—Ni1—N1	86.89 (7)	N2—C9—H9	118.6
Ni1—O1w—H1w1	118.7 (18)	C10—C9—H9	118.6
Ni1—O1w—H1w2	131.3 (16)	C11—C10—C9	119.5 (2)
H1w1—O1w—H1w2	102 (2)	C11—C10—H10	120.3
Ni1—O2w—H2w1	107.6 (18)	C9—C10—H10	120.3
Ni1—O2w—H2w2	111.2 (19)	C10—C11—C12	120.1 (2)
H2w1—O2w—H2w2	113 (3)	C10—C11—H11	120.0
Ni1—O3w—H3w1	119 (2)	C12—C11—H11	120.0
Ni1—O3w—H3w2	123.6 (19)	C11—C12—C13	116.6 (2)
H3w1—O3w—H3w2	104 (3)	C11—C12—C14	124.4 (2)
H4w1—O4w—H4w2	104 (3)	C13—C12—C14	118.9 (2)
H5w1—O5w—H5w2	104 (3)	N2—C13—C12	123.1 (2)
H6w1—O6w—H6w2	113 (3)	N2—C13—C17	117.12 (17)
C1—N1—Ni1	117.36 (13)	C12—C13—C17	119.8 (2)
C1—N1—H1	111.8 (16)	C15—C14—C12	121.3 (2)
Ni1—N1—H1	104.8 (16)	C15—C14—H14	119.3
C1—N1—H2	109.2 (16)	C12—C14—H14	119.3
Ni1—N1—H2	105.4 (16)	C14—C15—C16	121.1 (2)
H1—N1—H2	108 (2)	C14—C15—H15	119.5
C9—N2—C13	117.89 (18)	C16—C15—H15	119.5
C9—N2—Ni1	128.95 (15)	C17—C16—C18	116.6 (2)
C13—N2—Ni1	113.14 (13)	C17—C16—C15	119.0 (2)
C20—N3—C17	117.96 (18)	C18—C16—C15	124.4 (2)
C20—N3—Ni1	128.87 (14)	N3—C17—C16	123.43 (19)
C17—N3—Ni1	113.03 (13)	N3—C17—C13	116.75 (18)
C6—C1—C2	119.71 (18)	C16—C17—C13	119.82 (19)
C6—C1—N1	120.00 (17)	C19—C18—C16	119.7 (2)
C2—C1—N1	120.20 (17)	C19—C18—H18	120.1
C1—C2—C3	120.88 (18)	C16—C18—H18	120.1
C1—C2—H2A	119.6	C18—C19—C20	119.7 (2)
C3—C2—H2A	119.6	C18—C19—H19	120.1
C4—C3—C2	118.95 (18)	C20—C19—H19	120.1
C4—C3—C7	121.31 (18)	N3—C20—C19	122.5 (2)
C2—C3—C7	119.74 (17)	N3—C20—H20	118.7
C3—C4—C5	120.86 (18)	C19—C20—H20	118.7
C3—C4—H4	119.6		
			
N2—Ni1—N1—C1	63.44 (14)	C4—C5—C8—O4	176.82 (19)
O2w—Ni1—N1—C1	−110.83 (14)	C6—C5—C8—O4	−3.6 (3)
N3—Ni1—N1—C1	−16.38 (14)	C13—N2—C9—C10	−0.6 (3)
O3w—Ni1—N1—C1	154.68 (14)	Ni1—N2—C9—C10	−179.05 (18)
O1w—Ni1—N2—C9	−85.7 (2)	N2—C9—C10—C11	1.0 (4)
N3—Ni1—N2—C9	−177.4 (2)	C9—C10—C11—C12	0.5 (4)
O3w—Ni1—N2—C9	2.4 (2)	C10—C11—C12—C13	−2.2 (4)
N1—Ni1—N2—C9	89.4 (2)	C10—C11—C12—C14	178.1 (2)
O1w—Ni1—N2—C13	95.80 (15)	C9—N2—C13—C12	−1.2 (3)
N3—Ni1—N2—C13	4.16 (14)	Ni1—N2—C13—C12	177.41 (17)
O3w—Ni1—N2—C13	−176.05 (15)	C9—N2—C13—C17	178.95 (19)
N1—Ni1—N2—C13	−89.08 (15)	Ni1—N2—C13—C17	−2.4 (2)
O1w—Ni1—N3—C20	85.17 (19)	C11—C12—C13—N2	2.6 (3)
N2—Ni1—N3—C20	179.1 (2)	C14—C12—C13—N2	−177.6 (2)
O2w—Ni1—N3—C20	1.45 (19)	C11—C12—C13—C17	−177.6 (2)
N1—Ni1—N3—C20	−87.66 (19)	C14—C12—C13—C17	2.2 (3)
O1w—Ni1—N3—C17	−99.33 (15)	C11—C12—C14—C15	179.4 (3)
N2—Ni1—N3—C17	−5.39 (14)	C13—C12—C14—C15	−0.3 (4)
O2w—Ni1—N3—C17	176.95 (15)	C12—C14—C15—C16	−1.8 (5)
N1—Ni1—N3—C17	87.83 (15)	C14—C15—C16—C17	2.0 (4)
Ni1—N1—C1—C6	−93.21 (19)	C14—C15—C16—C18	−177.9 (3)
Ni1—N1—C1—C2	83.2 (2)	C20—N3—C17—C16	1.7 (3)
C6—C1—C2—C3	−0.1 (3)	Ni1—N3—C17—C16	−174.38 (18)
N1—C1—C2—C3	−176.57 (17)	C20—N3—C17—C13	−178.14 (19)
C1—C2—C3—C4	−0.1 (3)	Ni1—N3—C17—C13	5.8 (2)
C1—C2—C3—C7	179.01 (18)	C18—C16—C17—N3	0.0 (4)
C2—C3—C4—C5	−0.1 (3)	C15—C16—C17—N3	−179.9 (2)
C7—C3—C4—C5	−179.23 (18)	C18—C16—C17—C13	179.8 (2)
C3—C4—C5—C6	0.6 (3)	C15—C16—C17—C13	−0.1 (4)
C3—C4—C5—C8	−179.89 (18)	N2—C13—C17—N3	−2.3 (3)
C2—C1—C6—C5	0.5 (3)	C12—C13—C17—N3	177.8 (2)
N1—C1—C6—C5	177.01 (17)	N2—C13—C17—C16	177.8 (2)
C4—C5—C6—C1	−0.8 (3)	C12—C13—C17—C16	−2.0 (3)
C8—C5—C6—C1	179.70 (18)	C17—C16—C18—C19	−1.9 (4)
C4—C3—C7—O2	1.1 (3)	C15—C16—C18—C19	178.0 (3)
C2—C3—C7—O2	−177.98 (19)	C16—C18—C19—C20	2.0 (4)
C4—C3—C7—O1	−179.3 (2)	C17—N3—C20—C19	−1.5 (3)
C2—C3—C7—O1	1.7 (3)	Ni1—N3—C20—C19	173.77 (18)
C4—C5—C8—O3	−2.2 (3)	C18—C19—C20—N3	−0.3 (4)
C6—C5—C8—O3	177.34 (18)		

### Hydrogen-bond geometry (Å, °)

**Table:**

*D*—H···*A*	*D*—H	H···*A*	*D*···*A*	*D*—H···*A*
O1w—H1w1···O6w^i^	0.84 (1)	1.91 (1)	2.747 (2)	177 (3)
O1w—H1w2···O2^ii^	0.84 (1)	1.82 (1)	2.654 (2)	180 (2)
O2w—H2w1···O5w^i^	0.85 (1)	1.94 (1)	2.781 (2)	173 (3)
O2w—H2w2···O4^iii^	0.85 (1)	1.98 (1)	2.832 (2)	178 (3)
O3w—H3w1···O5w^iv^	0.84 (1)	2.16 (2)	2.914 (3)	148 (3)
O3w—H3w2···O3^ii^	0.85 (1)	1.88 (1)	2.721 (2)	173 (3)
O4w—H4w1···O6w^v^	0.85 (1)	1.97 (1)	2.817 (3)	171 (3)
O4w—H4w2···O2^iv^	0.85 (1)	2.16 (2)	2.915 (3)	149 (3)
O5w—H5w1···O1	0.85 (1)	1.90 (1)	2.726 (3)	163 (3)
O5w—H5w2···O3^vi^	0.84 (1)	1.91 (1)	2.716 (2)	159 (3)
O6w—H6w1···O1	0.85 (1)	1.83 (1)	2.678 (2)	177 (3)
O6w—H6w2···O4^iii^	0.85 (1)	1.95 (1)	2.791 (2)	176 (3)
N1—H1···O4w	0.85 (1)	2.08 (1)	2.928 (3)	173 (2)
N1—H2···O4^iii^	0.85 (1)	2.30 (1)	3.116 (2)	162 (2)

Symmetry codes: (i) −*x*+2, −*y*+1, −*z*+1; (ii) *x*−1/2, −*y*+3/2, *z*+1/2; (iii) −*x*+3/2, *y*−1/2, −*z*+1/2; (iv) *x*−1, *y*, *z*; (v) −*x*+3/2, *y*+1/2, −*z*+1/2; (vi) −*x*+5/2, *y*−1/2, −*z*+1/2.
